# The role of microflow patterns combined with greyscale ultrasound in enhancing diagnostic validity and reducing unnecessary biopsy rate of thyroid nodules

**DOI:** 10.1007/s00330-025-11963-w

**Published:** 2025-09-03

**Authors:** Wanying Li, Luying Gao, Yahong Wang, Min Zhang, Yiyan Du, Hongyan Wang, Jianchu Li

**Affiliations:** 1 Department of Ultrasound, State Key Laboratory of Complex Severe and Rare Diseases, Peking Union Medical College Hospital, Chinese Academy of Medical Sciences and Peking Union Medical College, Beijing, China; 2 Department of Ultrasound, Wuxi Branch of Ruijin Hospital Shanghai Jiao Tong University School of Medicine, Jiangsu, China

**Keywords:** Thyroid nodule, Ultrasound, Superb microvascular imaging, Diagnostic performance, Unnecessary biopsy rate

## Abstract

**Objective:**

To explore the value of microflow patterns based on superb microvascular imaging (SMI) combined with greyscale ultrasound in thyroid nodule diagnosis and biopsy recommendation.

**Materials and methods:**

Adult patients with thyroid nodules were recruited from May 2023 to February 2024. The greyscale features of nodules were evaluated according to the five ultrasound risk stratification systems (RSSs). The microflow patterns on SMI were used to adjust the category of nodules. The crab claw-like and the root hair-like patterns were malignant signs for upgrading, with the wheel-like and the arborescent patterns for downgrading. The diagnostic performance and biopsy recommendation of microflow patterns combined with greyscale ultrasound were analyzed.

**Results:**

A total of 253 nodules (136 malignant, 117 benign) in 203 patients were included. Chinese Thyroid Imaging Reporting and Data System (C-TIRADS) owned the best diagnostic sensitivity and the largest AUC, with no significant improvement for the former (0.853 vs 0.904, *p* = 0.096) but an evident increase for the latter (0.834 vs 0.900, *p* < 0.001) when combined with SMI. American College of Radiology (ACR) TI-RADS had the highest specificity, which was further enhanced by SMI (0.803 vs 0.855, *p* = 0.041). The unnecessary biopsy rates of RSSs were reduced by 1.27–20.30% according to microflow patterns on SMI. Among these, C-TIRADS had the lowest unnecessary biopsy rate and the largest reduction after the adjustment by SMI (38.04% vs 17.74%).

**Conclusion:**

The microflow patterns on SMI could enhance the diagnostic validity of greyscale ultrasound for thyroid nodules. Besides, the unnecessary biopsy rate could also be decreased by combining with SMI.

**Key Points:**

***Question*** *The utility of combining superb microvascular imaging (SMI) with greyscale ultrasound for the diagnosis of thyroid nodules and the recommendation of biopsies remains unclear.*

***Findings*** *Risk stratification systems adjusted by microflow patterns on SMI outperformed the original systems in distinguishing thyroid carcinoma and recommending biopsies.*

***Clinical relevance*** *Microflow patterns combined with greyscale ultrasound can enhance the diagnostic validity and reduce the unnecessary biopsy rate for thyroid nodules.*

**Graphical Abstract:**

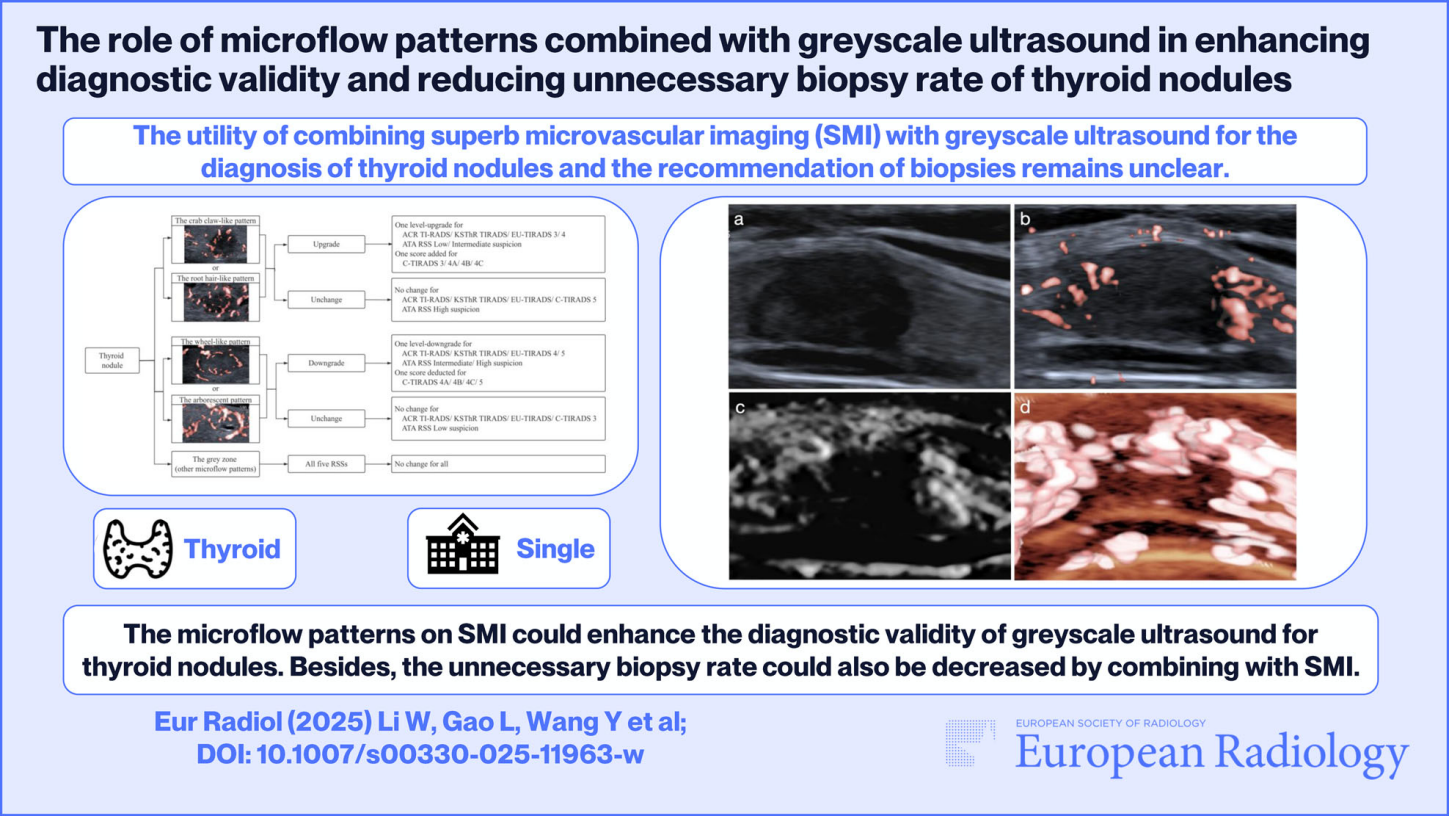

## Introduction

Recent years have witnessed a rise in the incidence of thyroid cancer, ranking as the seventh most prevalent cancer worldwide [1]. Though the overall prognosis of thyroid cancer is great, accurate preoperative diagnosis is crucial to the surgery decision and follow-up plan. With the growing understanding of the indolent nature of thyroid cancers [2], enhancing diagnostic accuracy but reducing the risk of overdiagnosis and overtreatment is of great clinical value.

Ultrasound is the preferred method for examining thyroid cancer. To solve the aforementioned problem, ultrasound risk stratification systems (RSSs) like Thyroid Imaging Reporting and Data System (TI-RADS) were developed to help evaluate the malignancy risk of thyroid nodules and establish criteria for biopsy by ultrasound. But these RSSs are based on greyscale ultrasound, and the specificity for recommending biopsy is low [3, 4].

Angiogenesis plays a vital role in tumor development, invasion, and metastasis [5]. However, the sensitivity of color Doppler flow imaging (CDFI) is far from satisfactory for depicting neovessels of thyroid cancer, and power Doppler flow imaging (PDFI) is limited by clutter and overflow when displaying minute vessels [6]. Superb microvascular imaging (SMI), as the new ultrasonic Doppler technology, owns superiority in tiny vessels and microflows with low velocity. Besides, it is convenient and noninvasive, which makes it possible for the vascular index to be widely used clinically. We first came up with the “microflow pattern” index on SMI to describe the architecture formed by the internal and peripheral microflow of thyroid nodules and confirmed that microflow patterns benefit the differentiation between benign and malignant thyroid nodules in our previous study [7]. However, the effect of microflow patterns combined with greyscale ultrasound, which is more suitable for clinical use, remains unknown.

Therefore, our study aimed to figure out the added value of SMI to greyscale ultrasound by comparing the diagnostic validity and unnecessary biopsy rate of commonly used RSSs before and after the combination with microflow patterns for thyroid nodules.

## Materials and methods

This study was approved by the Institutional Review Board of Peking Union Medical College Hospital (protocol number K3557). Written informed consent of all patients was obtained.

### Study participants

From May 2023 to February 2024, 300 consecutive patients with thyroid nodules in our center were prospectively recruited. The inclusion criteria were (1) aged 18 years or older, (2) preoperative conventional ultrasound and SMI examination were approved, (3) fine needle aspiration biopsy (FNAB) or surgical procedure was planned for pathological results, and (4) no history of thyroid surgery or preoperative treatment like thermal ablation therapy. The indications for FNAB were referred to the Chinese TI-RADS (C-TIRADS) [8] or the patients’ insistence. 97 cases were excluded for (1) loss to follow-up or canceled operation for personal reasons (*n* = 21), (2) the vascularity detection of the nodules near the cervical arteries was influenced by the arterial pulsation (*n* = 8), (3) entirely calcified nodules with posterior acoustic shadowing (*n* = 3), (4) failure of one-to-one correspondence between the ultrasound results and pathological results when multiple nodules existed (*n* = 9), (5) follicular lesions of undetermined significance or nodules of Bethesda grades I, III and IV (*n* = 56). Nodules of malignant pathological results after surgery or Bethesda V/VI after FNAB were included in the malignant group, and those with benign histopathological results or Bethesda II were included in the benign group.

### Image acquisition

One radiologist with 5 years of experience in thyroid ultrasound scanning performed the ultrasound examination for all the patients using Aplio 500 (Canon Medical Systems Corp.) equipped with a 5–14 MHz linear transducer or Aplio 800 (Canon Medical Systems Corp.) with a 5–18 MHz linear transducer. Patients were instructed to lie supine, tilt their head back to fully expose the examination area and breathe smoothly, avoiding swallowing as much as possible. Greyscale ultrasound was performed comprehensively to identify the thyroid nodules in transverse and longitudinal planes. Then SMI was used for vascular features with minimum pressure applied to the transducer to avoid vessel collapse. The region of interest was set to include the entire nodule with 3–5 mm of the surrounding parenchyma. The color gain was adjusted as high as possible to keep the microflows but suppress the background noise. Besides, the scale was set at 1.0–2.5 cm/s, frame rate 25–60 f/s, dynamic range 60 dB. Monochrome SMI (mSMI) predominated in demonstrating microflow patterns owing to the high sensitivity, while color SMI (cSMI) served for the position relation of microflows and nodules and distinguishing microflows from calcifications. Smart-3D was also constructed for the three-dimensional view of microflow patterns based on cSMI. Cine clips on greyscale ultrasound, mSMI and cSMI were saved.

### Data interpretation

Two radiologists with over 5 years of experience in thyroid ultrasound independently analyzed the videos and classified the nodules according to greyscale features. Disagreement was solved by consulting an expert radiologist with over 20 years of experience in thyroid ultrasound. The largest diameter was recorded as the size of the nodules. The location of nodules was classified as left lobe, right lobe and isthmus. We referred to the American College of Radiology (ACR) TI-RADS [9] for identifying greyscale features of nodules since it gave a comprehensive interpretation. Though RSSs varied, the suspicious features were similar, and we regarded solid composition, hypo- or marked hypoechogenicity, taller-than-wide shape, lobulated or irregular margin or extra-thyroidal extension and punctate echogenic foci as malignant greyscale features for thyroid nodules. Each nodule was graded according to the ACR TI-RADS [9], the American Thyroid Association (ATA) RSS [10], Korean Society of Thyroid Radiology (KSThR) TIRADS [11], European TI-RADS (EU-TIRADS) [12] and C-TIRADS [8], respectively. For nodules that were not classifiable by the ATA RSS, the intermediate suspicion category was given [13]. Biopsy recommendation of each nodule according to different RSSs was also recorded.

**Fig. 1:**
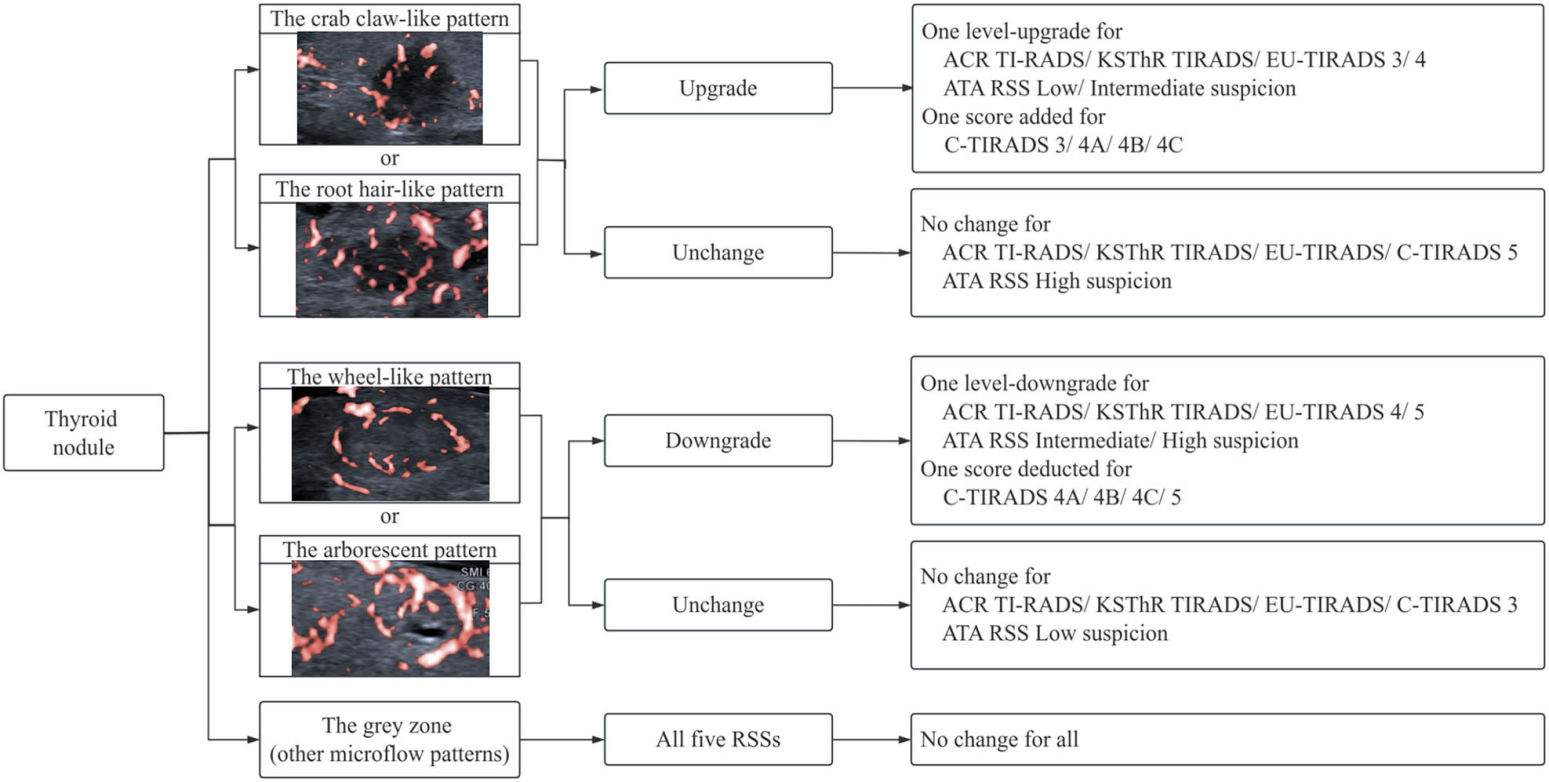
The flowchart of classification regulation by microflow patterns on SMI combined with greyscale ultrasound for thyroid nodules

Three radiologists with 10 years, 6 years, and 3 years of experience in thyroid ultrasound observed the microflow pattern based on SMI of nodules. The interpreted results were recorded separately, and the radiologists reviewed again for the microflow pattern 6 months later, blinded to the previous ultrasound and pathological results. According to the results of our previous research, the crab claw-like pattern and the root hair-like pattern were microflow characteristics of malignant thyroid nodules with the wheel-like pattern and the arborescent pattern of benign nodules [7]. Owing to this, we divided the microflow patterns into three types: the crab claw-like or the root hair-like pattern, the wheel-like or the arborescent pattern, and the gray zone (for which none of the aforementioned patterns could be assigned). The crab claw-like pattern was an aggregation of multiple penetrating vessels; the root hair-like pattern was a large penetrating vessel giving out small branches inside the nodule; the wheel-like pattern represented peripheral circumferential vessels with some branches inside the nodule; the arborescent pattern was a large branch from the circumferential vessel to the inside of the nodule with small branches [7]. Then we used the microflow pattern to adjust the grade of each nodule. For nodules with the crab claw-like pattern or the root hair-like pattern, a one-level upgrade was made for ACR TI-RADS, ATA RSS, KSThR TIRADS, EU-TIRADS, and one score was added for C-TIRADS. For nodules with the wheel-like pattern or the arborescent pattern, a one-level downgrade was made for ACR TI-RADS, ATA RSS, KSThR TIRADS, EU-TIRADS, and one score was deducted for C-TIRADS. The original grade remained still for nodules with a microflow pattern of gray zone. No upgrade was made for nodules of TR5 and high suspicion, with no downgrade for nodules of TR3 and low suspicion. The flowchart of classification regulation is shown in Fig. 1. Biopsy recommendation of each nodule with the adjusted grade according to different RSSs was also recorded.

### Statistical analysis

The Kolmogorov-Smirnov test was used for the normality of continuous variables. The age of patients and the size of nodules were continuous variables that did not form a normal distribution, and a median with interquartile range (IQR) was calculated. Categorical variables were expressed as frequencies. The location, greyscale features and microflow pattern of nodules in malignant and benign groups were compared by the $\chi$^2^ test, with the Mann-Whitney U test for the size of nodules. Univariate and multivariate analyses were used to test the value of microflow patterns. Collinearity existed if the Spearman correlation coefficient was 0.70 or larger, and the features would be carefully screened for multivariate logistic regression analysis. Cohen’s Kappa was used for intraobserver agreement of microflow patterns, and Fleiss’ Kappa was used for interobserver agreement, with values 0.01–0.20 slight agreement, 0.21–0.40 fair agreement, 0.41–0.60 moderate agreement, 0.61–0.80 substantial agreement and 0.81–0.99 almost perfect agreement [14]. Taking the pathological results for the gold standard, receiver operator characteristic (ROC) curves of greyscale ultrasound and greyscale ultrasound combined with SMI according to different RSSs in diagnosing thyroid nodules were drawn for the best cutoff value, and the diagnostic sensitivity, specificity, positive predictive value (PPV), negative predictive value (NPV) and accuracy were also calculated. The area under the ROC curve (AUC) of each RSS was compared with that of the RSS combined with SMI. The number of nodules recommended for biopsy according to five RSSs and the RSSs combined with SMI in the benign and malignant groups was counted. The proportion of the benign nodules recommended for biopsy in all the recommended nodules was the unnecessary biopsy rate [15]. All statistical tests were bilateral, and a *p*-value < 0.05 was considered statistically significant. The analyses were conducted by using SPSS software version 26.0 (SPSS Software Inc.), MedCalc software version 20.0 (MedCalc Software Ltd.) and GraphPad Prism Software version 8.0 (GraphPad Inc.).

## Results

### Demographic and pathology data

In this study, 203 patients with 253 thyroid nodules were included. The median age of patients was 45 years (IQR: 35–58 years), and the male-to-female ratio was 1:3.61. 165 patients underwent thyroid surgery, and 38 patients had FNAB only. Among the nodules with surgical pathology, 131 were papillary thyroid carcinoma, 63 were nodular goiter, 15 were thyroid adenoma, 6 were focal lymphocytic thyroiditis. Among the nodules with FNAB pathology only, 5 were of Bethesda VI and 33 were of Bethesda II. All the nodules of Bethesda V were malignant with surgical confirmation. In all, 136 nodules were included in the malignant group and 117 in the benign group.

### Ultrasound characteristics

**Fig. 2:**
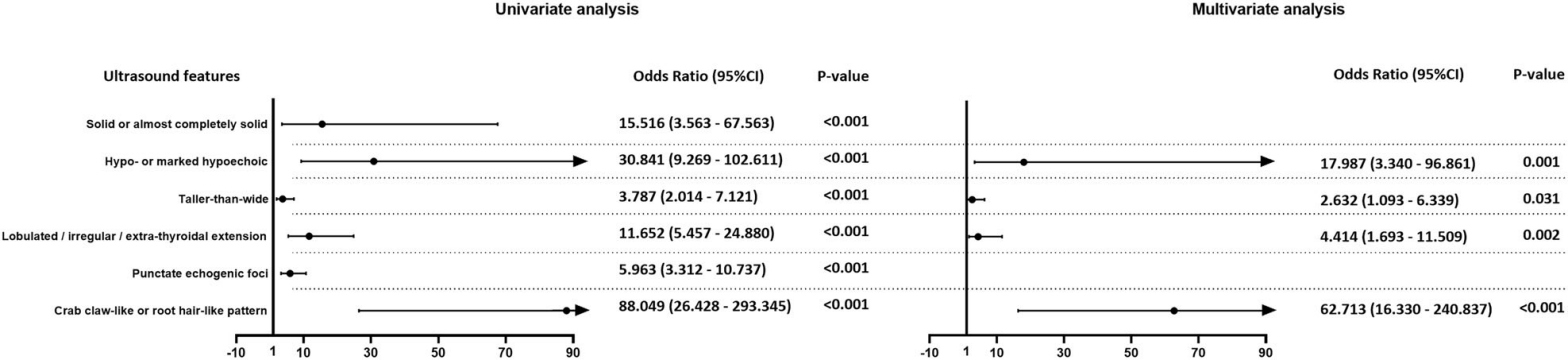
Forest plot of odds ratios of the ultrasound features in the univariate and multivariate analysis

The ultrasound features of thyroid nodules in the malignant and benign groups are compared in Table 1. All the features of nodules in the malignant and benign groups differed significantly (*p* < 0.05). Based on clinical experience, we included solid or almost completely solid composition, hypo- or marked hypoechogenicity, taller-than-wide shape, lobulated or irregular margin or extra-thyroidal extension, punctate echogenic foci, and the crab claw-like or the root hair-like pattern into the univariate and multivariate analysis (Fig. 2). Results showed that the crab claw-like or the root hair-like pattern was one independent risk factor for malignant thyroid nodules (odds ratio 62.713, 95% CI 16.330–240.837).

**Table 1:** Greyscale and SMI features of thyroid nodules in the malignant and benign groups

Feature	Malignant group (*n* = 136)	Benign group (*n* = 117)	*p*-value
Size (cm, IQR)	0.9 (0.7–1.2)	1.7 (0.9–3.05)	< 0.001
Location			0.027
Left lobe	55	59	
Right lobe	69	56	
Isthmus	12	2	
Composition			< 0.001
Solid or almost completely solid	134	95	
Mixed cystic and solid	2	22	
Echogenicity			< 0.001
Hypoechoic	123	67	
Marked hypoechoic	10	2	
Isoechoic	3	44	
Hyperechoic	0	4	
Shape			< 0.001
Taller-than-wide	51	16	
Wider-than-tall	85	101	
Margin			< 0.001
Lobulated or irregular	65	9	
Extra-thyroidal extension	2	0	
Ill-defined	34	9	
Smooth	35	99	
Echogenic foci			< 0.001
Punctate echogenic foci	72	20	
Peripheral calcifications	2	5	
Macrocalcifications	9	11	
Punctate echogenic foci + macrocalcifications	3	0	
None	50	81	
Microflow pattern			< 0.001
Crab claw-like pattern	70	3	< 0.001
Root hair-like pattern	25	0	< 0.001
Wheel-like pattern	4	60	< 0.001
Arborescent pattern	7	22	0.001
Gray zone	30	32	

*SMI* superb microvascular imaging, *IQR* interquartile range

Table 2 shows substantial or almost perfect intra- and interobserver agreements for microflow patterns based on SMI of thyroid nodules.

**Table 2:** The intra- and interobserver agreements among the microflow pattern of thyroid nodules

Microflow pattern	Intraobserver agreement (Cohen’s Kappa)			Interobserver agreement (Fleiss’ Kappa)
	Reviewer 1	Reviewer 2	Reviewer 3	
Crab claw-like pattern	0.809	0.896	0.781	0.735
Root hair-like pattern	0.930	0.920	0.694	0.724
Wheel-like pattern	0.947	0.978	0.808	0.893
Arborescent pattern	0.839	0.979	0.743	0.768
Phenotype	0.861	0.927	0.774	0.782

### The diagnostic performance of greyscale ultrasound before and after the combination with SMI

**Fig. 3:**
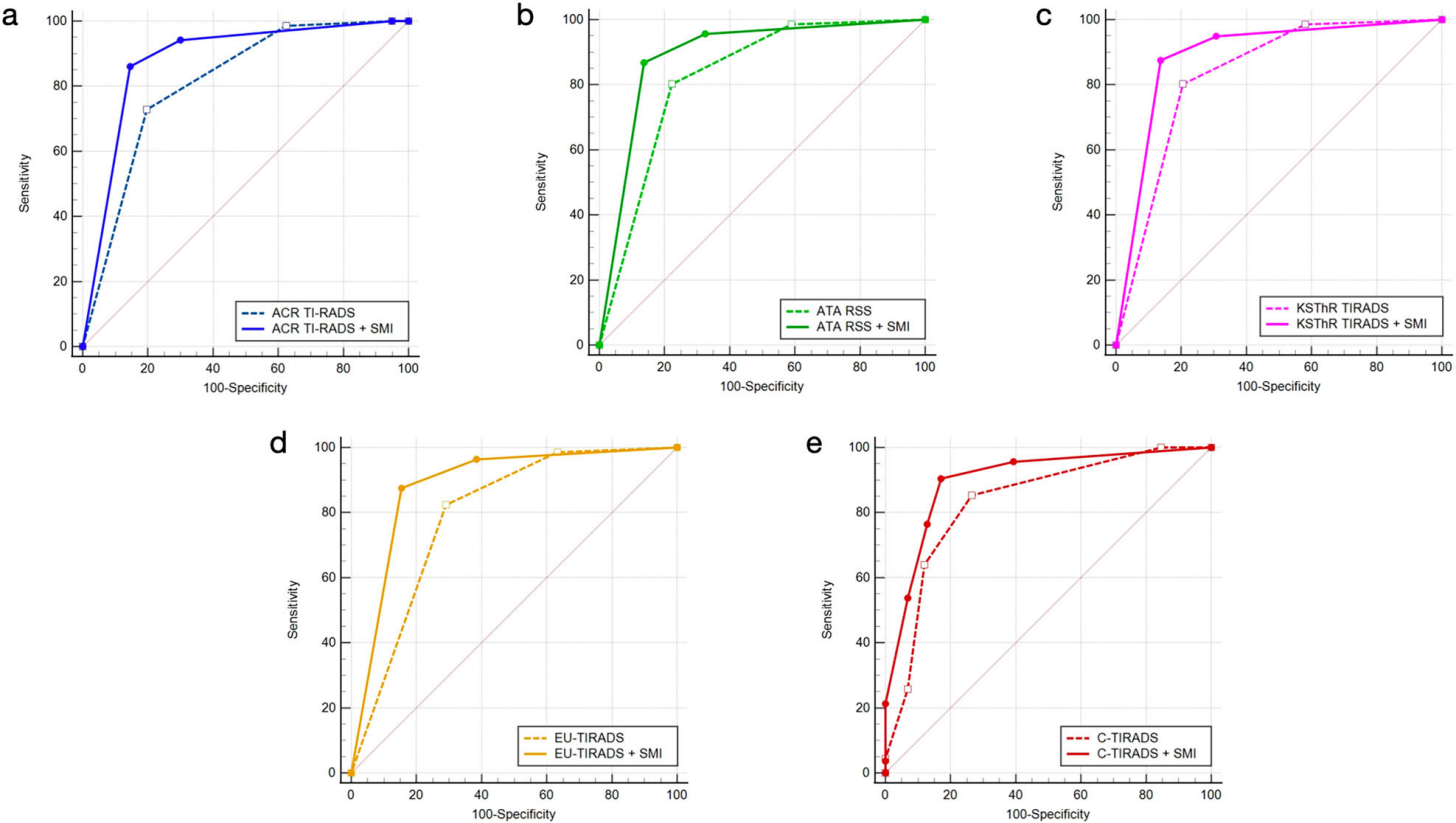
The ROC curves comparison of greyscale ultrasound and greyscale ultrasound combined with microflow patterns on SMI according to different RSSs. **a** ACR TI-RADS, **b** ATA RSS, **c** KSThR TIRADS, **d** EU-TIRADS, **e** C-TIRADS

The classification adjustments of thyroid nodules made by microflow patterns combined with greyscale ultrasound according to each RSS are summarized in Table 3. The diagnostic performances of greyscale ultrasound and greyscale ultrasound combined with microflow patterns on SMI according to different RSSs are shown in Table 4 and Fig. 3. After the combination with SMI, the diagnostic specificity of ACR TI-RADS (0.803 vs 0.855, *p* = 0.041), ATA RSS (0.778 vs 0.863, *p* = 0.004), KSThR TIRADS (0.795 vs 0.863, *p* = 0.013), EU-TIRADS (0.709 vs 0.846, *p* < 0.001) and C-TIRADS (0.735 vs 0.829, *p* = 0.006) in differentiating benign and malignant thyroid nodules improved significantly with the sensitivity of ACR TI-RADS (0.728 vs 0.860, *p* = 0.001) and KSThR TIRADS (0.801 vs 0.875, *p* = 0.034) increased significantly. The AUCs of greyscale ultrasound combined with SMI according to all five RSSs were higher than those of the greyscale ultrasound alone (*p* < 0.05).

**Table 3:** Grade adjustment of thyroid nodules by microflow patterns on SMI

RSS	Adjustment	Number of adjusted nodules	Original grade	Adjusted grade	Malignant nodules	Benign nodules
ACR TI-RADS	Upgrade	23	TR3	TR4	0	2
			TR4	TR5	21	0
	Downgrade	55	TR4	TR3	6	40
			TR5	TR4	3	6
ATA RSS	Upgrade	16	Low suspicion	Intermediate suspicion	0	2
			Intermediate suspicion	High suspicion	14	0
	Downgrade	52	Intermediate suspicion	Low suspicion	4	33
			High suspicion	Intermediate suspicion	5	10
KSThR TI-RADS	Upgrade	16	TR3	TR4	0	2
			TR4	TR5	14	0
	Downgrade	51	TR4	TR3	5	34
			TR5	TR4	4	8
EU-TIRADS	Upgrade	15	TR3	TR4	0	2
			TR4	TR5	13	0
	Downgrade	56	TR4	TR3	3	31
			TR5	TR4	6	16
C-TIRADS	Upgrade	54	TR4A	TR4B	10	1
			TR4B	TR4C	18	2
			TR4C	TR5	23	0
	Downgrade	76	TR4A	TR3	6	53
			TR4B	TR4A	3	12
			TR4C	TR4B	1	1

*SMI* superb microvascular imaging, *RSS* risk stratification system, *ACR TI-RADS* American College of Radiology Thyroid Imaging Reporting and Data System, *ATA* American Thyroid Association, *KSThR TIRADS* Korean Society of Thyroid Radiology Thyroid Imaging Reporting and Data System, *EU-TIRADS* European Thyroid Imaging Reporting and Data System, *C-TIRADS* Chinese Thyroid Imaging Reporting and Data System

**Table 4:** Diagnostic performances of greyscale ultrasound according to different RSSs and RSSs combined with SMI

Diagnostic method	Cutoff value	Sensitivity	*p*-value	Specificity	*p*-value	PPV	NPV	Accuracy	AUC (95% CI)	*p*-value
ACR TI-RADS	TR5	0.728	0.001	0.803	0.041	0.811	0.718	0.763	0.811 (0.758–0.858)	< 0.001
ACR TI-RADS + SMI	TR5	0.860		0.855		0.873	0.840	0.858	0.883 (0.837–0.920)	
ATA RSS	High Suspicion	0.801	0.066	0.778	0.004	0.807	0.771	0.791	0.825 (0.772–0.869)	< 0.001
ATA RSS + SMI	High Suspicion	0.868		0.863		0.881	0.849	0.866	0.891 (0.846–0.927)	
KSThR TIRADS	TR5	0.801	0.034	0.795	0.013	0.820	0.775	0.798	0.834 (0.782–0.878)	< 0.001
KSThR TIRADS + SMI	TR5	0.875		0.863		0.881	0.856	0.870	0.890 (0.845–0.926)	
EU-TIRADS	TR5	0.824	0.169	0.709	< 0.001	0.767	0.776	0.771	0.794 (0.739–0.842)	< 0.001
EU-TIRADS + SMI	TR5	0.875		0.846		0.869	0.853	0.862	0.883 (0.837–0.920)	
C-TIRADS	TR4B	0.853	0.096	0.735	0.006	0.789	0.811	0.798	0.834 (0.782–0.878)	< 0.001
C-TIRADS + SMI	TR4B	0.904		0.829		0.860	0.882	0.870	0.900 (0.857–0.934)	

*RSS* risk stratification system, *SMI* superb microvascular imaging, *PPV* positive predictive value, *NPV* negative predictive value, *AUC* area under the receiver operator characteristic curve, *CI* confidence interval, *ACR TI-RADS* American College of Radiology Thyroid Imaging Reporting and Data System, *ATA* American Thyroid Association, *KSThR TIRADS* Korean Society of Thyroid Radiology Thyroid Imaging Reporting and Data System, *EU-TIRADS* European Thyroid Imaging Reporting and Data System, *C-TIRADS* Chinese Thyroid Imaging Reporting and Data System

### The biopsy recommendation of greyscale ultrasound before and after the combination with SMI

**Fig. 4:**
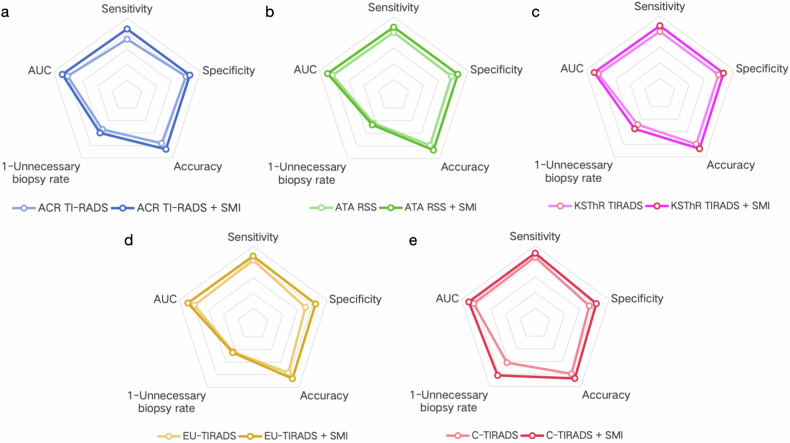
Radar graphs of the diagnostic and biopsy recommending performances of greyscale ultrasound and greyscale ultrasound combined with microflow patterns on SMI according to different RSSs. **a** ACR TI-RADS, **b** ATA RSS, **c** KSThR TIRADS, **d** EU-TIRADS, **e** C-TIRADS

**Fig. 5:**
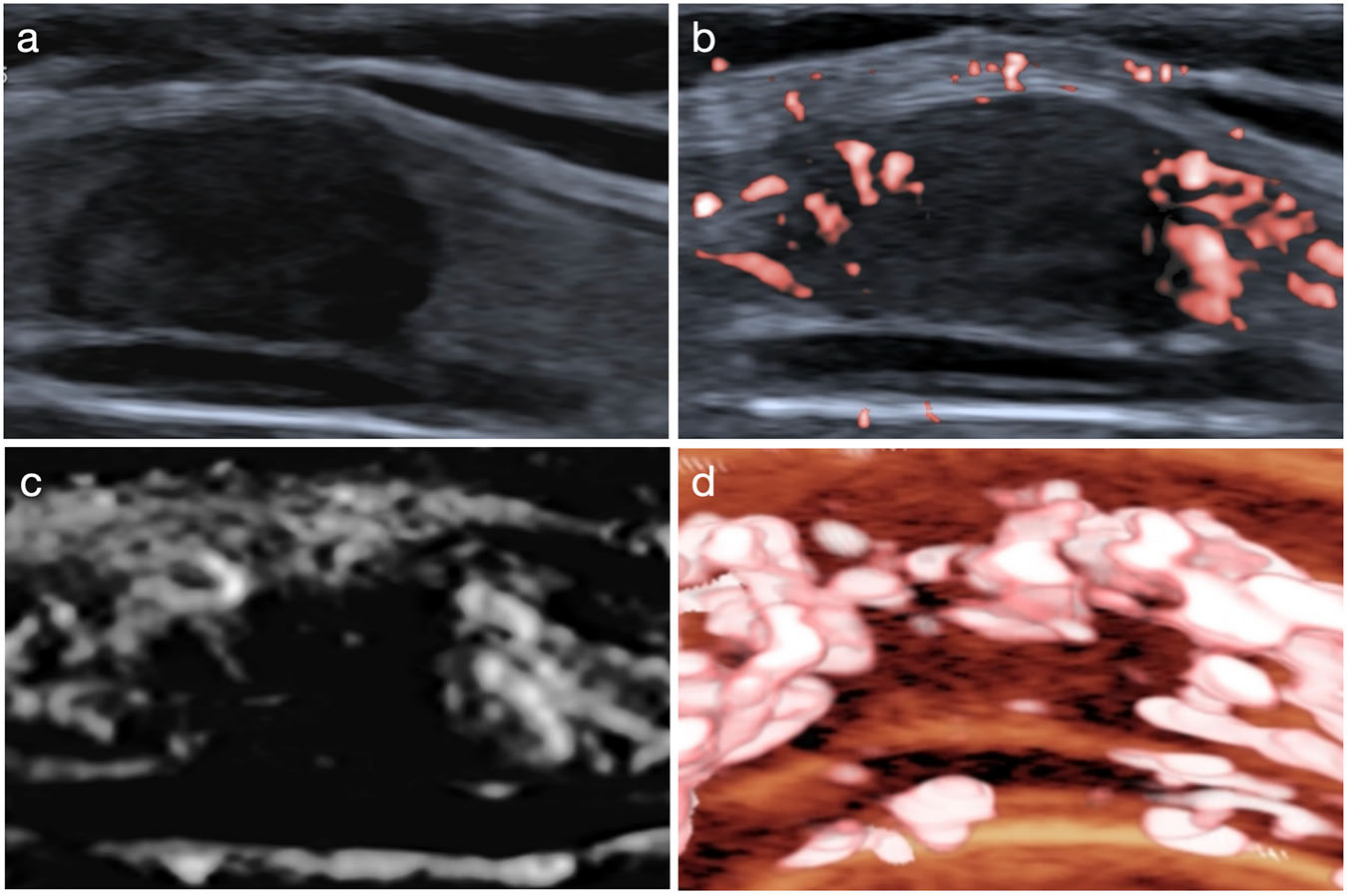
Ultrasound images of a solid hypoechoic nodule of 1.1 cm in the thyroid isthmus of a 41-year-old female patient. After adjustment by the crab claw-like microflow pattern on SMI, one-level upgrade was made for ACR TI-RADS, ATA RSS, KSThR TIRADS, EU-TIRADS and C-TIRADS, and the biopsy recommendations of ACR TI-RADS and EU-TIRADS were added. Postoperative pathology confirmed it was a papillary thyroid carcinoma. **a** Greyscale ultrasound, **b** CSMI, **c** MSMI, **d** Smart-3D

**Fig. 6:**
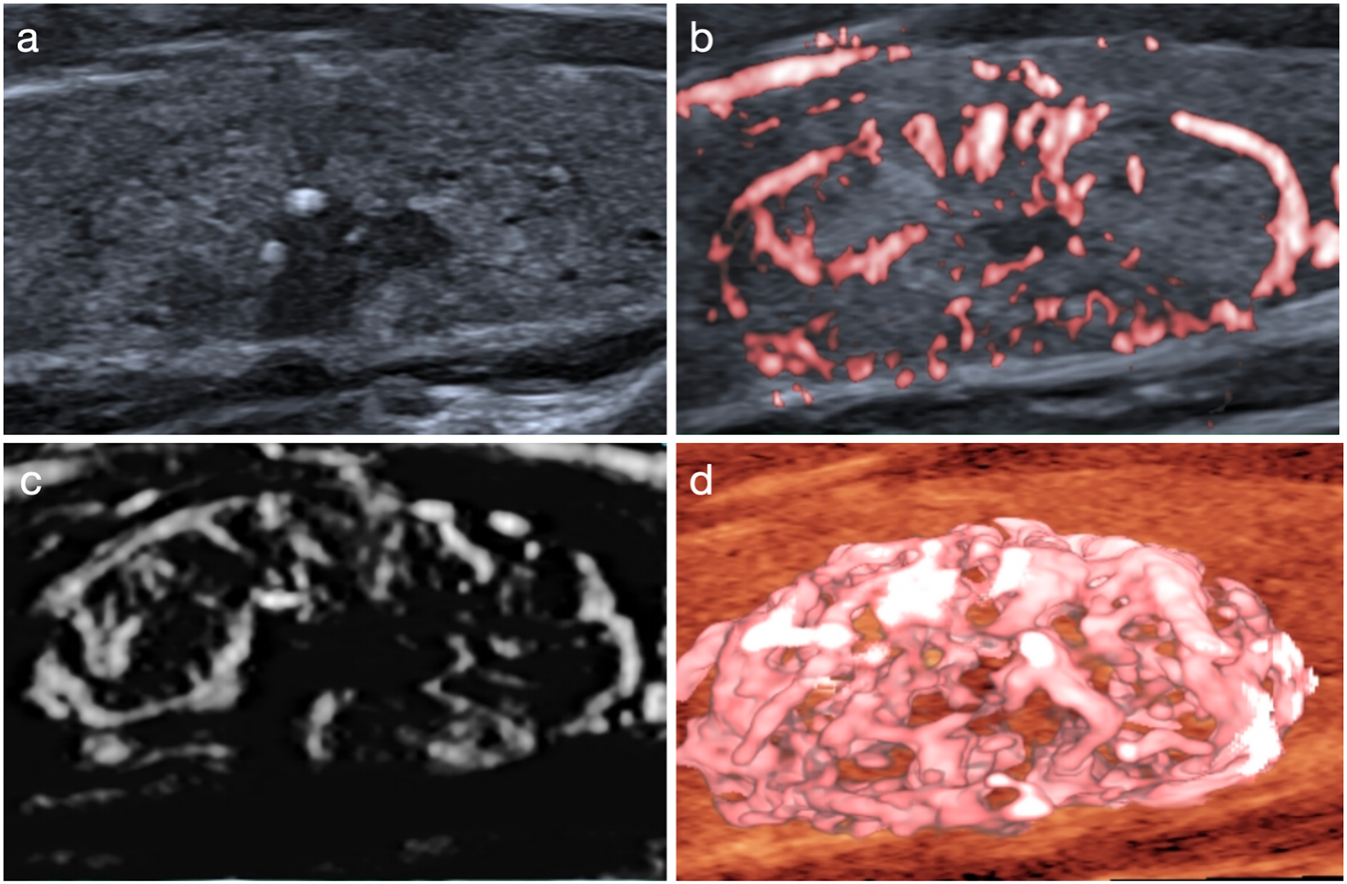
Ultrasound images of a solid isoechoic nodule of 2.0 cm with punctate echogenic foci in the right thyroid lobe of a 60-year-old female patient. After adjustment by the wheel-like microflow pattern on SMI, one-level downgrade was made for ACR TI-RADS, ATA RSS, KSThR TIRADS, EU-TIRADS and C-TIRADS, and the biopsy recommendations of ACR TI-RADS and KSThR TIRADS were exempted. Cytopathological result showed a benign nodule of Bethesda II. **a** Greyscale ultrasound, **b** CSMI, **c** MSMI, **d** Smart-3D

Biopsy recommendations of greyscale ultrasound and greyscale ultrasound combined with microflow patterns on SMI according to different RSSs are summarized in Table 5. Compared with using RSSs alone, the microflow patterns combined with the ACR TI-RADS, ATA RSS, KSThR TIRADS, EU-TIRADS and C-TIRADS reduced the unnecessary biopsy rate by 5.48%, 2.61%, 6.75%, 1.27% and 20.30% respectively. The diagnostic and biopsy recommending performances of greyscale ultrasound before and after combining with microflow patterns on SMI according to different RSSs are summarized in Fig. 4 and illustrated by examples in Figs. 5, 6 and Supplementary Appendix Figs. 1, 2.

**Table 5:** Biopsy recommendation of different RSSs and RSSs combined with SMI

Recommendation method	Recommended nodules in the malignant group	Recommended nodules in the benign group	Unnecessary biopsy rate
ACR TI-RADS	71	60	45.80%
ACR TI-RADS + SMI	74	50	40.32%
ATA RSS	63	80	55.94%
ATA RSS + SMI	63	72	53.33%
KSThR TIRADS	71	75	51.37%
KSThR TIRADS + SMI	72	58	44.62%
EU-TIRADS	51	66	56.41%
EU-TIRADS + SMI	48	59	55.14%
C-TIRADS	101	62	38.04%
C-TIRADS + SMI	102	22	17.74%

*RSS* risk stratification system, *SMI* superb microvascular imaging, *ACR TI-RADS* American College of Radiology Thyroid Imaging Reporting and Data System, *ATA* American Thyroid Association, *KSThR TIRADS* Korean Society of Thyroid Radiology Thyroid Imaging Reporting and Data System, *EU-TIRADS* European Thyroid Imaging Reporting and Data System, *C-TIRADS* Chinese Thyroid Imaging Reporting and Data System

## Discussion

The study explored the value of microflow patterns on SMI combined with greyscale ultrasound in thyroid nodule diagnosis and biopsy recommendations according to five ultrasound RSSs for thyroid nodules. After classification adjustment by SMI, the diagnostic performance of ACR TI-RADS, ATA RSS, KSThR TIRADS, EU-TIRADS, and C-TIRADS improved in differentiation between benign and malignant thyroid nodules compared to the use of RSSs only. The unnecessary biopsy rate recommended by each RSS also decreased after the combination with microflow patterns.

Our results showed that microflow patterns assisted in the differentiation between benign and malignant thyroid nodules. Recent years have witnessed a heated debate on the role of vascularity in diagnosing thyroid nodules by ultrasound. Some scholars found vascularity exerted a positive influence [16, 17], others believed no added value was applied to the conventional ultrasound [18–20]. Owing to this, vascular indices have not been included in any RSSs of ultrasound for the assessment of thyroid nodules. The advent of SMI brought the argument to a climax. Compared to the CDFI and PDFI, SMI is more sensitive to the tiny vessels and microflows with low velocity, thus better depicts the vascularity of thyroid nodules. Previous research mainly focused on the vascular richness, morphology and distribution on SMI, and the conclusions varied [6, 21–24]. We attributed this controversy to the interobserver agreement and different definitions, and brought out the microflow patterns to distinguish the benign and malignant thyroid nodules with substantial intra- and interobserver agreement [7]. Our results showed that the crab claw-like or the root hair-like pattern had similar diagnostic value as the widely-acknowledged malignant ultrasound features like taller-than-wide shape and lobulated or irregular margin, which served as the foundation for the SMI combined with greyscale ultrasound in the differentiation between benign and malignant thyroid nodules.

In our study, the addition of SMI enhanced the diagnostic validity of greyscale ultrasound. One meta-analysis included 39 studies demonstrated that the most accurate risk category thresholds for malignant thyroid nodules were TR5 for ACR TI-RADS, KSThR TIRADS, EU-TIRADS and high suspicion for ATA RSS [25], which were similar to our results. After adjustment by SMI, the AUCs increased significantly regardless of RSSs, showing a promising added value of SMI to the greyscale ultrasound. In comparison between the RSSs, ACR TI-RADS had a higher specificity but was less sensitive than ATA RSS and KSThR TIRADS [15, 26, 27]. Our results showed a significant improvement was made in the diagnostic sensitivity for ACR TI-RADS and the diagnostic specificity for all five RSSs by SMI, which offset the weaknesses. There were studies combining SMI with greyscale ultrasound in thyroid nodules, but the conclusions differed. No statistical difference was found after the use of SMI to greyscale ultrasound in the study of Ahn et al [28] and Yoon et al [29]. However, Zhao et al [30] and Zhu et al [31] believed the diagnostic performance improved by SMI combined with greyscale ultrasound. Chen et al [32] thought the added value of SMI existed in ACR TI-RADS 4 nodules. As far as we are concerned, the diagnostic criteria of SMI for malignant thyroid nodules and the methods that SMI combined with greyscale ultrasound counted a lot. The benign and malignant SMI features varied among these studies, some of which even went the opposite [28, 29]. In our study, we used microflow patterns for the first time to divide the SMI features into three categories: the crab claw-like or the root hair-like pattern, the wheel-like or the arborescent pattern, and the gray zone for the convenience of clinical application. And we kept the original classification rules of RSSs for the same reason. Meta-analysis research of SMI combined with greyscale ultrasound showed that the summary sensitivity, specificity and AUC in diagnosing thyroid nodules were 0.80–0.88, 0.79–0.89 and 0.89–0.94 [33, 34]. However, no subgroup analysis was performed according to the diagnostic criteria of SMI. More studies are needed for the standardization of SMI diagnostic criteria to examine the added value of SMI.

We found that SMI combined with greyscale ultrasound helped in reducing unnecessary biopsy rate. With the growing concerns regarding the overdiagnosis and overtreatment of thyroid nodules, active surveillance has been gradually recognized as the primary management strategy for low-risk nodules [35, 36]. Under such a background, refining biopsy decision-making has emerged as another critical research focus alongside improving diagnostic performance, while the unnecessary biopsy rate is receiving increasing attention. Ha et al [37] found the unnecessary biopsy rate of ACR TI-RADS was 25.3%, much lower than the ATA RSS (51.7%) and KSThR TIRADS (56.9%). As for C-TIRADS, we found the unnecessary biopsy rate was the lowest, followed by ACR TI-RADS, which was similar to the results of Jin et al [38]. And the unnecessary biopsy rate of C-TIRADS could be further reduced by more than half after combining with SMI in our study, with varying degrees of decrease of other RSSs. The decision of biopsy was based on the size as well as the classification of the nodules. In this study, we maintained the size criteria but regulated the biopsy recommendation according to the adjusted classification by microflow patterns on SMI. A few studies have made similar attempts with SMI to improve the biopsy recommendation. Ahn et al [28] found that the combined method of KSThR TIRADS, SMI and strain elastography had better performance in the management decision of biopsy. But the added value of SMI alone was unclear in this study. Ren et al [39] constructed a radiomics model of greyscale ultrasound combined with SMI for thyroid nodules and discovered a significant decrease in unnecessary biopsy rate of ACR TI-RADS. Our study discovered a general decline in the unnecessary biopsy rates among different RSSs. Besides, the results showed no reduction in the number of malignant nodules recommended for biopsy by microflow patterns combined with RSSs, except for EU-TIRADS. Considering the indications for biopsy of thyroid nodules are required to detect the highest possible percentage of thyroid malignancy while minimizing unnecessary biopsies [37], we thought the microflow patterns combined with greyscale ultrasound could mitigate the risk of overtreatment to a certain extent.

Our study had several limitations. First of all, selection bias existed. The study was conducted in a tertiary hospital, and we only included patients with FNAB or surgery, so a large portion of benign nodules could not be observed, which led to a relatively high prevalence of malignant nodules in our study. This also induced the differences in nodule size between the benign and malignant groups since the biopsy size criteria increased with the decrease of the grade. And we did not include thyroid nodules of Bethesda categories I, III, and IV without repeated FNAB for no definite pathology results. So the diagnostic value of SMI for these nodules remains unknown. Second, the diagnosis of some benign nodules was based on cytopathology, which might lead to the false-negative result. Third, subgroup analysis according to nodule size was not made, but unnecessary biopsy rates of RSSs were influenced by nodule size cutoff [15]. Finally, the outcome of this study was based on a single center with single examination operator and fixed ultrasound settings to keep the consistency. But this also influenced the universality of our results. Therefore, external validation of a larger sample size is necessary.

In conclusion, this study preliminarily demonstrated that microflow patterns on SMI could improve the diagnostic performance of greyscale ultrasound in the differentiation between benign and malignant thyroid nodules. What’s more, the added application of microflow patterns could reduce the unnecessary biopsy rates of ultrasound RSSs.

## Supplementary information

ELECTRONIC SUPPLEMENTARY MATERIAL

## Abbreviations

ACR: American College of Radiology
ATA: American Thyroid Association
AUC: Area under the curve
CDFI: Color Doppler flow imaging
cSMI: Color SMI
C-TIRADS: Chinese Thyroid Imaging Reporting and Data System
EU-TIRADS: European Thyroid Imaging Reporting and Data System
FNAB: Fine needle aspiration biopsy
IQR: Interquartile range
KSThR: Korean Society of Thyroid Radiology
mSMI: Monochrome SMI
NPV: Negative predictive value
PPV: Positive predictive value
ROC: Receiver operator characteristic
RSS: Risk Stratification System
SMI: Superb microvascular imaging
TI-RADS: Thyroid Imaging Reporting and Data System
