# Supplementary material for: The role of microflow patterns combined with greyscale ultrasound in enhancing diagnostic validity and reducing unnecessary biopsy rate of thyroid nodules
Source: Eur Radiol. 2025 Sep 3;36(3):1771–82. doi: 10.1007/s00330-025-11963-w (PMC12963240; doi:10.1007/s00330-025-11963-w)
Supplement: Supplementary file 1 — ELECTRONIC SUPPLEMENTARY MATERIAL [file 330_2025_11963_MOESM1_ESM.pdf]

The role of microflow patterns combined with greyscale ultrasound in enhancing diagnostic validity and reducing unnecessary biopsy rate of thyroid nodules

ELECTRONIC SUPPLEMENTARY MATERIAL

Supplemental appendix figure 1

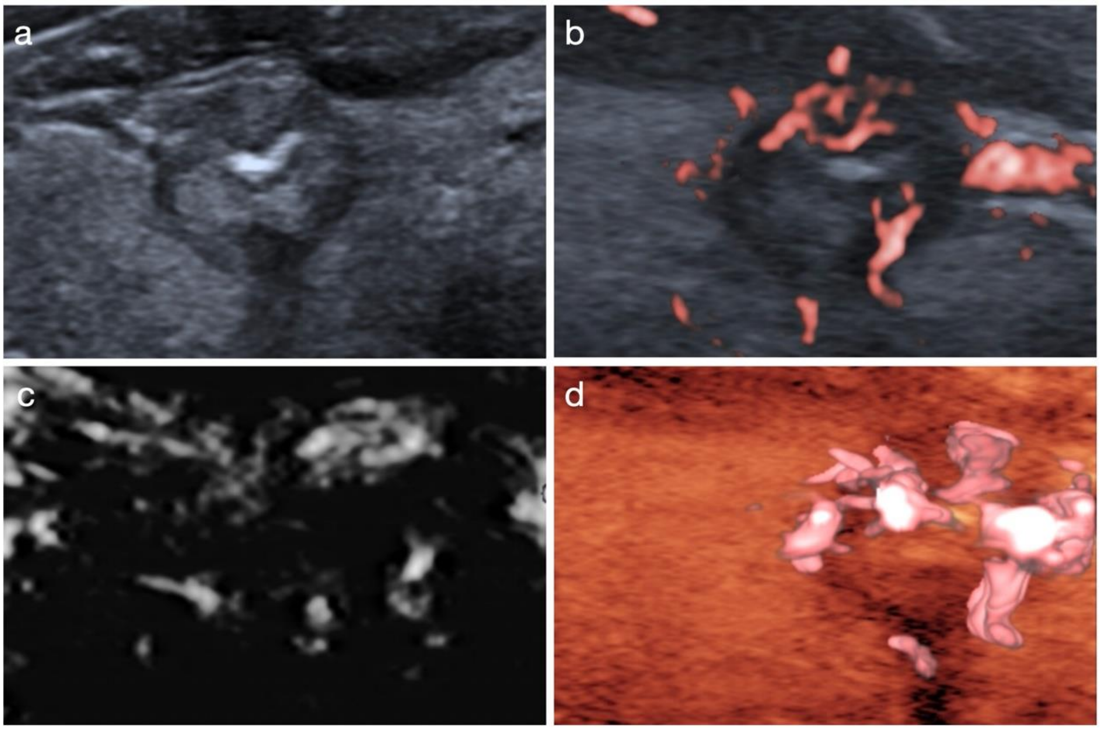

Ultrasound images of a solid hypoechoic nodule with macrocalcification of 0.9 cm in the right thyroid lobe of a 44-year-old female patient. After adjustment by the root hair-like microflow pattern on SMI, one level upgrade was made for ACR TI-RADS, ATA RSS, KSThR TIRADS, EU-TIRADS and C-TIRADS, and the biopsy recommendation of C-TIRADS was added. Postoperative pathology confirmed it was a papillary thyroid carcinoma. (a) Greyscale ultrasound (b) CSMI (c) MSMI (d) Smart-3D.

Supplemental appendix figure 2

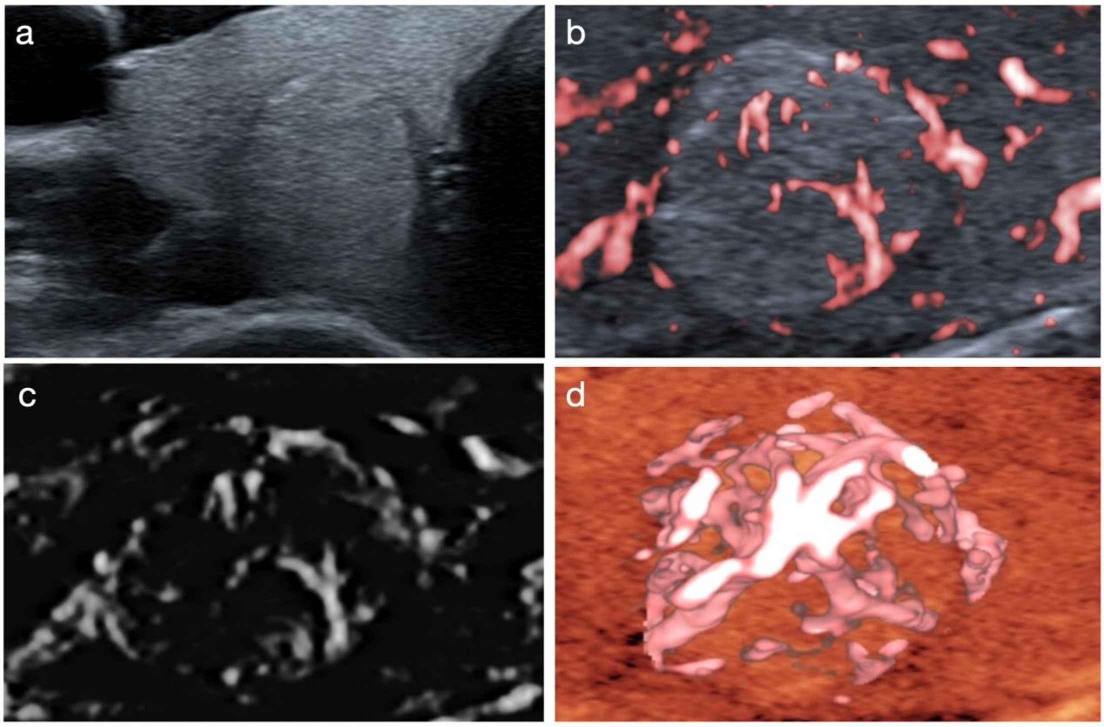

Ultrasound images of a solid isoechoic nodule with a taller-than-wide shape of 1.2 cm in the right thyroid lobe of a 42-year-old female patient. After adjustment by the arborescent microflow pattern on SMI, one level downgrade was made for ACR TI-RADS, ATA RSS, KSThR TIRADS, EU-TIRADS and C-TIRADS, and the biopsy recommendations of ATA RSS, EU-TIRADS and C-TIRADS were exempted. Cytopathological result showed a benign nodule of Bethesda II. (a) Greyscale ultrasound (b) CSMI (c) MSMI (d) Smart-3D.
